# Prognostic Value of Three Different Methods of *MGMT* Promoter Methylation Analysis in a Prospective Trial on Newly Diagnosed Glioblastoma

**DOI:** 10.1371/journal.pone.0033449

**Published:** 2012-03-13

**Authors:** Arne Christians, Christian Hartmann, Axel Benner, Jochen Meyer, Andreas von Deimling, Michael Weller, Wolfgang Wick, Markus Weiler

**Affiliations:** 1 Clinical Cooperation Unit Neuropathology, German Cancer Research Center (DKFZ), Heidelberg, Germany; 2 Clinical Cooperation Unit Neurooncology, German Cancer Research Center (DKFZ), Heidelberg, Germany; 3 Division of Biostatistics, German Cancer Research Center (DKFZ), Heidelberg, Germany; 4 Department of Neuropathology, Institute of Pathology, Heidelberg University Hospital, Heidelberg, Germany; 5 Department of Neurooncology at the National Center for Tumour Diseases, Heidelberg University Hospital, Heidelberg, Germany; 6 Department of Neurology, University Hospital Zurich, Zurich, Switzerland; 7 Department of General Neurology, Hertie Institute for Clinical Brain Research, University of Tübingen, Tübingen, Germany; 8 Department of Neuropathology, Institute of Pathology, Hannover Medical School (MHH), Hannover, Germany; University of Navarra, Spain

## Abstract

Hypermethylation in the promoter region of the *MGMT* gene encoding the DNA repair protein O^6^-methylguanine-DNA methyltransferase is among the most important prognostic factors for patients with glioblastoma and predicts response to treatment with alkylating agents like temozolomide. Hence, the *MGMT* status is widely determined in most clinical trials and frequently requested in routine diagnostics of glioblastoma. Since various different techniques are available for *MGMT* promoter methylation analysis, a generally accepted consensus as to the most suitable diagnostic method remains an unmet need. Here, we assessed methylation-specific polymerase chain reaction (MSP) as a qualitative and semi-quantitative method, pyrosequencing (PSQ) as a quantitative method, and methylation-specific multiplex ligation-dependent probe amplification (MS-MLPA) as a semi-quantitative method in a series of 35 formalin-fixed, paraffin-embedded glioblastoma tissues derived from patients treated in a prospective clinical phase II trial that tested up-front chemoradiotherapy with dose-intensified temozolomide (UKT-05). Our goal was to determine which of these three diagnostic methods provides the most accurate prediction of progression-free survival (PFS). The *MGMT* promoter methylation status was assessable by each method in almost all cases (*n* = 33/35 for MSP; *n* = 35/35 for PSQ; *n* = 34/35 for MS-MLPA). We were able to calculate significant cut-points for the continuous methylation signals at each CpG site analysed by PSQ (range, 11.5 to 44.9%) and at one CpG site assessed by MS-MLPA (3.6%) indicating that a dichotomisation of continuous methylation data as a prerequisite for comparative survival analyses is feasible. Our results show that, unlike MS-MLPA, MSP and PSQ provide a significant improvement of predicting PFS compared with established clinical prognostic factors alone (likelihood ratio tests: *p*<0.001). Conclusively, taking into consideration prognostic value, cost effectiveness and ease of use, we recommend pyrosequencing for analyses of *MGMT* promoter methylation in high-throughput settings and MSP for clinical routine diagnostics with low sample numbers.

## Introduction

Assessment of the methylation status of the *O^6^-methylguanine-DNA methyltransferase* (*MGMT*) gene promoter in malignant glioma has become one of the most requested molecular assays in clinical neuro-oncology. Since the landmark study by Hegi *et al.* [1] numerous clinical trials in glioblastoma have confirmed that hypermethylation of the *MGMT* promoter serves as a strong prognostic factor for progression-free survival (PFS) and overall survival (OS) [1], [2], [3], [4], [5], [6]. The *MGMT* gene encodes a ubiquitously expressed suicide DNA repair enzyme that counteracts the normally lethal effects of alkylating agents by removing alkyl adducts from the O^6^-position of guanine [7]. O^6^-alkylated guanine causes base mispairing and double-strand breaks, thus inducing apoptosis and cell death [8]. Due to this DNA repair activity, the MGMT protein is believed to provide resistance against cytotoxic effects of alkylating agents [9]. This therapeutically disadvantageous protective effect is thought not to be present when *MGMT* is epigenetically silenced through promoter methylation as observed in many human cancers including glioblastoma, thus rendering cells more sensitive to alkylating drugs. Due to the prognostic and predictive role of the *MGMT* promoter status for patients suffering from malignant glioma, promoter methylation of this gene is commonly assessed both in clinical trials and routine diagnostics [10].

Several diagnostic methods are available for promoter methylation analysis: The most commonly used technique is methylation-specific polymerase chain reaction (MSP) [11], a non-quantitative method established for *MGMT* by Esteller *et al.* [2]. Other techniques of methylation analysis include real-time quantitative MSP (RT-MSP) [12], methylation-specific multiplex ligation-dependent probe amplification (MS-MLPA) [13], bisulfite sequencing [14], combined bisulfite restriction analysis (COBRA) [15], pyrosequencing (PSQ) [16], SIRPH (SNuPE ion pair-reverse phase high-performance liquid chromatography) [17], and others. Alternative methods of determining the *MGMT* status of a tumour include quantification of mRNA expression by quantitative reverse transcription polymerase chain reaction (qRT-PCR) [18], protein detection by immunohistochemistry (IHC) [19], [20], and assessment of MGMT activity [21], rather than promoter methylation analysis. Despite this variety of available techniques, a generally accepted consensus as to the most suitable method of assessing *MGMT* promoter methylation in glioma tissues has not been achieved so far, neither for the requirements of large clinical trials nor for routine diagnostics [10], [22].

In the present study, we analysed formalin-fixed, paraffin-embedded (FFPE) tumour specimens derived from patients with newly diagnosed glioblastoma who were treated up-front according to a dose-intensified TMZ-containing chemoradiotherapy protocol within a prospective clinical phase II trial (UKT-05) [5]. We compared three different assays for *MGMT* promoter methylation analysis and included MSP as a qualitative and potentially semi-quantitative, PSQ as a quantitative and MS-MLPA as a semi-quantitative method to determine which of these methods would predict clinical outcome most reliably.

## Results

### Analyses of *MGMT* promoter methylation by MSP, PSQ and MS-MLPA

**Figure 1:**
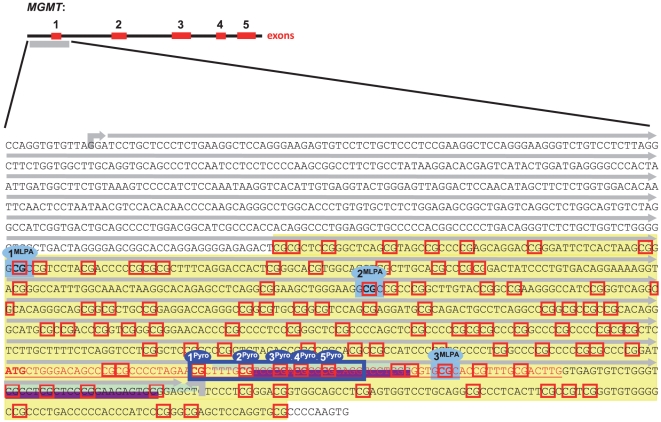
Schematic overview of the *MGMT* promoter region including CpG sites interrogated by each diagnostic method. Grey arrow, *MGMT* promoter region; filled yellow box, complete CpG island; red boxes, single CpG sites; understriked red labelled sequence, *MGMT* exon 1 with start codon marked in bold type; purple bars, methylation-specific (M) primers for MSP according to [2]; mint bars, unmethylation-specific (U) primers for MSP according to [2]; dark blue box, PSQ region comprising five CpG sites; light blue boxes, three GCGC *Hha*l sites for MS-MLPA.

The main goal of this study was to compare three different methods of *MGMT* promoter methylation assessment with regard to their respective value of predicting clinical outcome. To this end, we investigated methylation of the *MGMT* promoter by MSP, PSQ and MS-MLPA in 35 FFPE glioblastoma tissues derived from patients treated with dose-intensified TMZ in a prospective clinical phase II trial (UKT-05) [5]. A schematic overview of the *MGMT* promoter region including highlighted CpG sites addressed by each of the three diagnostic methods is given in Figure 1. Methylation data were successfully obtained in 33 of 35 (MSP), 35 of 35 (PSQ) and 34 of 35 (MS-MLPA) tumour specimens, respectively. The results of the three different diagnostic methods are listed in Table 1. For MSP, 14 of 33 analysed tumours (42%) were methylation-positive. As an alternative approach to evaluate the MSP data in a semi-quantitative way, the ratio of methylation was calculated for each specimen by comparing the intensities of methylated (M) and unmethylated (U) MSP bands. 7 of 33 analysed tumours displayed an M/U ratio>1 and were therefore assessed as strongly methylated, whereas for 11 of 33 tumours, an M/U ratio between 0 and 1 was calculated, indicating only weak *MGMT* promoter methylation. In 15 tumours, the M/U ratio was 0 (no M primer MSP product detectable), indicating an unmethylated *MGMT* promoter status. For PSQ, single methylation signals ranged from 0% to 100%. Averaged over all five CpGs, 15 of 35 tissues (43%) showed a mean methylation signal above 10%. Of these, 12 specimens (34%) showed a mean methylation signal above 30%. For MS-MLPA, methylation signals ranged between −25% and +76%. Averaged over all three CpG sites, 13/34 tissues (38%) displayed a mean methylation signal above 10%, and of these, 3 specimens (9%) above 30%.

**Table 1:** Results of *MGMT* promoter methylation assessed by MSP (qualitative, +/−, and semi-quantitative, M/U ratio), PSQ and MS-MLPA.

	MSP	PSQ	MS-MLPA
**Variable type**	binary	continuous	continuous
**Successful analysis, ** ***n*** ** (%)**	33/35 (94)	35/35 (100)	34/35 (97)
**Methylation +, ** ***n*** ** (%)**	14/33 (42)	N/A	N/A
**Methylation −, ** ***n*** ** (%)**	19/33 (58)	N/A	N/A
**M/U ratio>1, ** ***n*** ** (%)**	7/33 (21)	N/A	N/A
**M/U ratio<1, ** ***n*** ** (%)**	11/33 (33)	N/A	N/A
**M/U ratio = 0, ** ***n*** ** (%)**	15/33 (45)	N/A	N/A
**Mean methylation >10%, ** ***n*** ** (%)**	N/A	15/35 (43)	13/34 (38)
**Mean methylation >30%, ** ***n*** ** (%)**	N/A	12/35 (34)	3/34 (9)

For PSQ and MS-MLPA, the mean methylation signal averaged over all CpGs addressed in each method is indicated. N/A, not applicable.

### Cut-point testing and survival analyses

To allow for a direct comparison with the qualitative methylation data obtained by MSP in respect to survival, the continuous (semi-)quantitative methylation signals from PSQ and MS-MLPA were converted into binary methylation data. To this end, we used maximally selected log-rank statistics to test for cut-points in the continuous CpG methylation data obtained by PSQ and MS-MLPA and to estimate the corresponding cut-off value in case of significance. With respect to PFS, we identified significant cut-points for the quantitative methylation signals of all five CpG sites addressed by PSQ, and for MS-MLPA site 1. With respect to OS, cut-point estimations yielded a significant value only for PSQ CpG site 5 but for none of the interrogated MS-MLPA sites (Table 2). Notably, the cut-points for the PSQ CpG sites 1, 3 and 4 were in the range of 11.5%–14.0%. This correlates well with our observation that in normal brain tissue, the degree of *MGMT* promoter methylation mostly ranges between 0% and 10% (data not shown). For PSQ CpG 2 (PFS, 31.5%) and CpG 5 (PFS, 44.9%; OS, 44.3%) higher cut-points were calculated.

**Table 2:** Estimated cut-points for PSQ and MS-MLPA.

	Progression-free survival		Overall survival	
PSQ	Cut-point [%]	*p*	Cut-point [%]	*p*
**CpG 1**	11.8	<0.001	7.2	0.07
**CpG 2**	31.5	<0.001	3.9	0.06
**CpG 3**	14.0	<0.001	11.9	0.08
**CpG 4**	11.5	<0.001	11.5	0.18
**CpG 5**	44.9	<0.001	44.3	0.03

MS-MLPA	Cut-point [%]	*p*	Cut-point [%]	*p*
**site 1**	3.6	0.002	11.2	0.07
**site 2**	10.4	0.06	6.7	0.10
**site 3**	12.8	0.99	12.8	0.82

**Figure 2:**
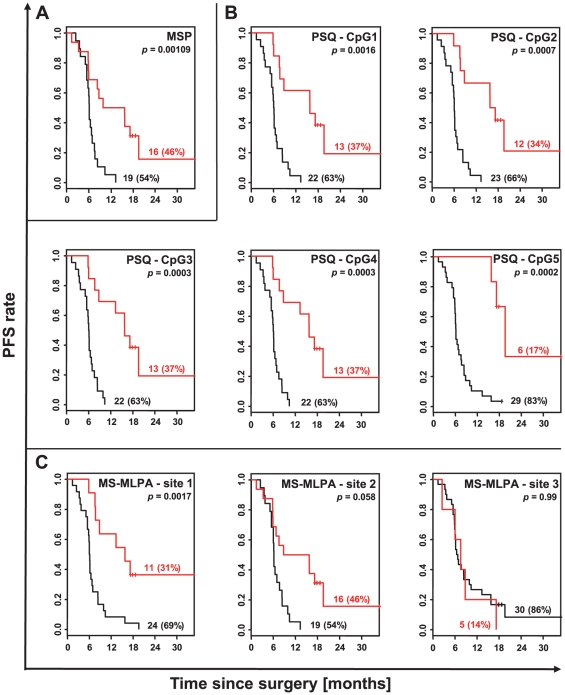
Method-dependent Kaplan-Meier estimates of PFS. PFS data for binary methylation covariates were obtained from (**A**) MSP, (**B**) PSQ CpG sites 1 to 5 and (**C**) MS-MLPA sites 1 to 3 based on missing data imputation (*n* = 35) for each diagnostic method applied. Respective cut-points allowing the conversion from continuous to binary methylation data in (B) and (C) are indicated in Table 2. Of note, Kaplan-Meier curves for MS-MLPA sites 2 and 3 were computed on grounds of insignificant cut-points (see Table 2). Respective *p* values of the maximally selected log-rank test and respective numbers (percentages) of tumours assigned to each group, methylated or unmethylated, are given in each plot. Black curves, unmethylated; red curves, methylated.

Significant cut-point values were then used to dichotomise the continuous methylation data from PSQ and MS-MLPA and generate Kaplan-Meier plots based on the binary covariates. Figure 2A–C provides plots of the Kaplan-Meier survival curve estimates for both qualitative MSP results and dichotomised methylation data from all five PSQ CpG sites and all three MS-MLPA sites. Of note, PSQ variables and particularly the resulting binary PSQ covariates were highly significant prognostic factors, whereas for MS-MLPA weaker associations were obtained. Applying semi-quantitative assessment of MSP based on M/U ratios, PFS was only significantly prolonged in patients suffering from tumours with strong promoter methylation (M/U ratio>1) when compared with all other patients (*p* = 0.008, log-rank test). However, tumour samples with an M/U ratio>1 were not significantly higher correlated with prolonged survival when specifically compared to tumours with few methylation, i.e. with an M/U ratio between 0 and 1 (PFS, *p* = 0.06; OS, *p* = 0.22, log-rank test). Moreover, semi-quantitative MSP using M/U ratios was not significantly associated with OS (*p* = 0.22, log-rank test). As opposed to this, conventionally applied qualitative MSP assessment (+/−) was superior in that it was significantly related to both survival endpoints, PFS (*p*<0.001; log-rank test) and OS (*p* = 0.04, log-rank test).

### Likelihood ratio tests and prediction errors

We determined the additional predictive value gained by the results of each methylation measurement relative to major therapy-independent clinical covariates for newly diagnosed glioblastoma, i.e., age, gender, Karnofsky performance status, extent of resection, using likelihood ratio tests (Table 3). MSP and PSQ improved prediction of PFS to a similar extent (*p*<0.001 for MSP and continuous and dichotomised PSQ data) while MS-MLPA did not provide a significant gain of additional information (*p* = 0.02). With regard to OS, only MSP delivered a significant gain of prediction relative to clinical data alone (*p* = 0.001) while the continuous data for both PSQ and MS-MLPA did not provide such an improvement (Table 3). Of note, it must be conceded that the likelihood ratio test results are overoptimistic as cut-point estimation was performed on the same data set. To this end, Table 3 additionally lists the cross-validated measures of prediction error and R^2^ that take this problem into account and hence show less optimistic values.

**Table 3:** *P* values of likelihood ratio tests (LR; clinical *vs.* clinical plus methylation data), apparent error (AE), 10-fold cross-validated prediction error (PE), and explained variation R^2^ (PE based R^2^ value), computed for 18-months follow-up.

	Progression-free survival				Overall survival			
Prediction Model	LR	AE	PE	R^2^	LR	AE	PE	R^2^
**Kaplan-Meier**	N/A	0.165	0.174	Ref.	N/A	0.162	0.172	Ref.
**Clinical Factors (CF)**	Ref.	0.164	0.209	−0.198	Ref.	0.133	0.172	−0.004
**CF + MSP**	< 0.001	0.118	0.152	0.128	0.001	0.107	0.145	0.155
**CF + PSQ (CpG 1–5)**	< 0.001	0.098	0.165	0.051	0.16	0.110	0.183	−0.066
**CF + dPSQ (CpG 1–5)**	< 0.001	0.076	0.153	0.124	N/A			
**CF + MS-MLPA (site 1–3)**	0.02	0.130	0.180	−0.032	0.03	0.112	0.172	−0.001
**CF + dMS-MLPA (site 1–3)**	N/A				N/A			

For LR, the clinical prognostic factors were used as a reference (Ref.). For R^2^, Kaplan-Meier was used as a reference (Ref.). MSP, methylation-specific polymerase chain reaction; dPSQ (CpG 1–5), dichotomised pyrosequencing data for CpG 1–5; dMS-MLPA, dichotomised methylation-specific multiplex ligation-dependent probe amplification data for site 1–3. N/A, not applicable.

**Figure 3:**
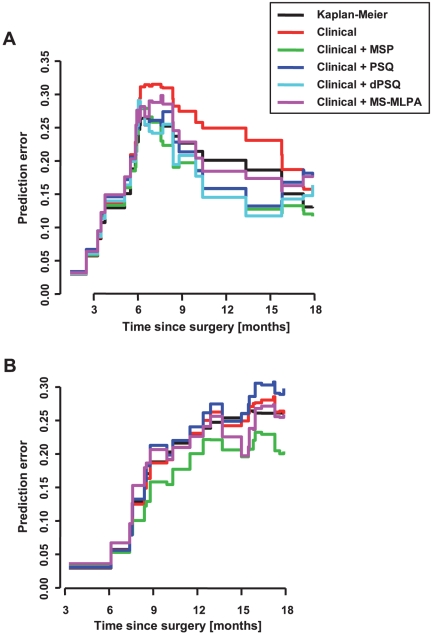
Prediction error curves for each diagnostic method with respect to (A) PFS and (B) OS. To assess the predictive accuracy of models including methylation data, the cumulating prediction error curves over 18 months follow-up time and a time-dependent R^2^-like measure were computed for the marginal Kaplan-Meier estimates (Kaplan-Meier), the Cox model using clinical data only (i.e., age, gender, Karnofsky performance status, extent of resection [Clinical]), and the Cox model using combined clinical plus methylation data as determined by MSP (Clinical + MSP), PSQ CpG sites 1 to 5 (Clinical + PSQ), dichotomised PSQ CpG sites 1 to 5 (Clinical + dPSQ), and MS-MLPA sites 1 to 3 (Clinical + MS-MLPA). Prediction error curves for dichotomised methylation data at MS-MLPA sites 1 to 3 (PFS and OS) and pyrosequencing CpG sites 1 to 5 (OS) were not feasible due to insignificant cut-points (see Table 2).

To assess the predictive accuracy of the three diagnostic methods relative to the above-stated clinical prognostic factors, we computed cumulative prediction error curves over 18 months follow-up time (which was near to the median follow-up of 21.7 months) and a time-dependent R^2^-like measure using the Kaplan-Meier model as a reference (Figure 3). With regard to PFS, we found that Kaplan-Meier estimates improved the survival predictability relative to sole clinical covariates, and both MSP and PSQ (both original and dichotomised data), in combination with the clinical covariates, tended to improve this effect to a similar degree. However, MS-MLPA plus clinical data hardly reduced the prediction error of the Kaplan-Meier reference (Figure 3A) reflecting the relative inferiority of this method as proved by the likelihood ratio tests (Table 3). With respect to OS, neither PSQ nor MS-MLPA data additionally decreased the prediction error of the Kaplan-Meier reference to a relevant extent, whereas MSP demonstrated a clear-cut improvement of OS prediction (Figure 3B).

## Discussion

Due to its high prognostic and predictive relevance, assessment of the *MGMT* status has become state-of-the-art in current and planned clinical trials in glioma as a prognosticator, to stratify patients or even to limit trial entry accordingly. Moreover, it is frequently requested in routine diagnostics as a prognostic tool. The ongoing debate among neuropathologists about whether or not *MGMT* testing should be incorporated into the next revision of the WHO classification scheme for central nervous system tumours also points at an emerging diagnostic role for *MGMT*. Meanwhile, an internationally accepted consensus as to the most appropriate diagnostic instrument for *MGMT* testing is still unmet [22].

In this study, we sought to clarify which of three frequently applied techniques of assessing *MGMT* promoter methylation, MSP, PSQ and MS-MLPA, would predict clinical outcome most accurately. We chose a well-characterized, uniform patient cohort newly diagnosed with glioblastoma that received upfront chemoradiotherapy with dose-intensified temozolomide within the UKT-05 trial [5]. We used PFS as the clinical outcome measure in order to avoid a potential bias caused by differences in post-progression treatments. Moreover, this endpoint reflected the primary endpoint in the original UKT-05 trial [5] and was recently validated by a study that proposed six-month PFS as an alternative primary efficacy endpoint to OS in newly diagnosed glioblastoma patients receiving temozolomide [23].

Although done on a comparatively low sample number, our study features the following distinctive methodological strengths: i) all tissues examined stemmed from a prospective multicentre trial; ii) all tissues were exclusively obtained from open tumour resections (either complete or partial) to avoid a tissue contamination bias and gate out the lacking therapeutic and prognostic effect of sole biopsies from our analysis; iii) all three diagnostic methods applied were performed at one neuropathological institute; iv) all three techniques were performed on FFPE tissues closely resembling the clinical routine situation.

The major findings of our study are: i) all three techniques tested are feasible on FFPE tissue; ii) a statistical dichotomisation of continuous methylation data obtained by PSQ is feasible within a given cohort and allows condensation to qualitative +/− results as a prerequisite for comparative survival analyses; iii) MS-MLPA-derived methylation data are correlated to a weaker degree than PSQ data, and the estimation of a valid cut-off value is probably more difficult with MS-MLPA than with PSQ, especially when smaller cohorts such as in clinical phase II trials are evaluated; iv) both MSP and PSQ are superior to MS-MLPA in predicting clinical outcome.

The *MGMT* gene located on chromosome 10q26 has five exons and a CpG-rich island of 762 bp with 98 CpG dinucleotides encompassing the first exon and large parts of the promoter (Figure 1). Despite a few studies that conducted correlative analyses between *MGMT* promoter methylation at individual CpG sites and gene expression [24], [25], [26], [27], it still remains an open question which or how many CpGs in the *MGMT* promoter CpG island (CGI) have a major impact on expression and best reflect response to treatment and survival.

Using comprehensive pyrosequencing of the entire *MGMT* CGI, mRNA expression analyses and luciferase reporter assays, a recent contribution to this issue identified a distinct region within *MGMT* exon 1 (spanning CpG73-90) and particularly four specific CpG sites (CpG 83, 86, 87 and 89) to be most critical in the transcriptional control of *MGMT* and thus recommendable for *MGMT* testing [28]. This region harbours the annealing sites of the most commonly used MSP primers (forward M primer, CpG76-80; forward U primer, CpG75-80; reverse M and U primers, CpG84-87) that were also applied in our study, as well as the five adjacent CpGs that we interrogated by PSQ (CpG74-78) (Figure 1). The fact that MSP and PSQ resulted in similar prediction of PFS may be a consequence of this partial overlap in analysed CpGs. Unlike MSP and PSQ, the three CpGs addressed by MS-MLPA are widespread throughout most of the *MGMT* CGI (CpG 9, 23 and 81) and two of them (CpG 9 and 23) are even located in the upstream promoter region, distant from the sites that were interrogated by MSP and PSQ (Figure 1). This might explain the weaker correlation of CpG methylation of the MS-MLPA data and their inferior value in predicting PFS in our study.

Recently published studies correlated the *MGMT* status with clinical survival data: Based on promoter-wide methylation analyses of snap-frozen glioblastoma tissues using quantitative bisulfite sequencing and correlations with mRNA expression, protein expression and PFS, Shah *et al.* proposed a new classification scheme using methylation data from three different regions of the entire *MGMT* promoter, and provided confirmative data achieved by MS-MLPA. This approach seems promising in that it accounts for whole promoter-wide methylation patterns and integrates methylation, expression and survival data [29].

In a prospective study examining 63 patients diagnosed with malignant glioma, Kreth *et al.* identified *MGMT* mRNA expression as a predictor of clinical outcome independent from *MGMT* promoter methylation underscoring the necessity of approaching MGMT biology more comprehensively and also elucidating methylation-independent mechanisms that may regulate MGMT expression [30]. Assessing the specimens of the UKT-05 trial for *MGMT* mRNA expression would have been tempting. However, only FFPE tissues were available from different sources that do not allow sufficient RNA extraction.

The value of PSQ, as shown in our analyses, is supported by Karayan-Tapon *et al.* who evaluated *MGMT* promoter methylation assessment by MSP, semi-quantitative MSP, PSQ, qRT-PCR, and IHC for their value of predicting OS in glioblastoma patients. In this study, PSQ reached the highest predictive value, particularly at CpG site 4 [31].

MSP has evolved as the “gold standard” for methylation analysis of the *MGMT* gene promoter. It is the easiest to perform and least expensive of the three methods and does not require any special equipment or consumables aside from what is present in most medical laboratories anyway. MSP is often regarded to be non-optimal for some settings, especially when performed on low quality DNA extracted from FFPE tissue [10], [26]. Irregular mosaic methylation patterns and incomplete bisulfite conversion may lead to mispriming and lower sensitivity and specificity [10], [26], [31], [32]. However, in our setting the assay was successful in a high percentage (94%). Other studies have reported a much higher failure rate of the MSP assay when FFPE tissue was used [1], [31]. In our setting, it turned out to be similarly good as PSQ as to the prediction of PFS and the only of the three assays that significantly improved prediction of OS. Although the MSP assay delivers, when functional, mostly easy-to-interpret results ready to assist clinical decision-making, its major downside consists in its inability to detect heterogeneous patterns of methylation. The use of the MSP technique as a semi-quantitative assay through the comparison of the relative intensities of M and U primer-specific MSP bands did not improve on the prognostic value of the conventional qualitative assessment of MSP. We conclude from this that MSP should be used as a purely qualitative assay that is less appropriate to allow (semi-)quantitative interpretations.

PSQ overcomes this problem as it provides quantitative information on the extent of methylation at each individual CpG site with high sensitivity and specificity [32]. However, the interpretation of PSQ data is limited by a lack of consensus concerning a biologically relevant threshold that allows conversion of the original continuous data into a clinically practical binary code, i.e., methylated or unmethylated. The results of our study indeed demonstrate that, within a given cohort of samples, such a dichotomisation of PSQ data is statistically feasible and allows comparative survival analyses leading to highly significant results. However, due to the high costs of the required equipment, PSQ is not widely used in clinical diagnostics when single samples are subject to analysis [26].

MS-MLPA is a semi-quantitative method that has the advantage of omitting a DNA-modifying bisulfite treatment step, thus avoiding additional damage to the sample DNA. Besides its requirement for special equipment and expensive reagents, MS-MLPA is limited by its dependence on the presence of *Hha*I restriction sites, and only one of the three *MGMT* CpG sites suitable for MS-MLPA is located within the region that is commonly analysed by MSP or PSQ (Figure 1). Furthermore, similar to PSQ, MS-MLPA data need an algorithm of conversion into a +/− code. In our study, such a dichotomisation was possible only for MS-MLPA site 1. However, MS-MLPA data did not improve prediction of survival significantly (Table 3, Figure 3).

Conclusively, taking into consideration the ability to predict clinical outcome, cost effectiveness and ease of use, pyrosequencing seems to be most suitable for methylation analysis in a high-throughput setting (e.g., for the evaluation of the *MGMT* status of many specimens in larger clinical trials) while MSP seems to be more convenient in clinical routine diagnostics when low numbers of specimens need to be examined at a time.

## Materials and Methods

### Clinical data

UKT-05 was designed as a prospective clinical phase II trial that included 41 adult patients (median Karnofsky performance status: 90%; median age: 56 years) who were newly diagnosed with glioblastoma and treated up-front according to a dose-intensified TMZ-containing chemoradiotherapy protocol. The ethics committee at the University of Tübingen (Tübingen, Germany) approved the trial (253/2004). All patients gave written informed consent prior to study entry. MGMT analyses are specifically mentioned in the informed consent. All patients also consented to this translational research to be performed. TMZ was administered orally before and after radiation therapy in a weekly alternating schedule starting at 150 mg/m^2^ on days 1–7 of 14 day-cycles (“1 week on/1 week off”), with individual dose adjustments of TMZ in 25 mg-steps according to weekly haemograms. Standard involved-field radiotherapy was delivered in daily single fractions of 1.8 to 2.0 Gy, 5 days per week. During radiotherapy, low-dose TMZ was given concomitantly at 50 mg/m^2^. In addition, maintenance indomethacin was orally administered at 25 mg twice daily throughout the entire treatment without individual dose adjustments. PFS was defined as the time interval between the day of surgery and tumour progression on magnetic resonance imaging according to the criteria of MacDonald *et al.* [33] and/or clinical progression. OS was defined as the time interval between the day of surgery and death. A more detailed description of this trial is given in [5]. The tumour specimens of 35 patients (85%) were assessable for *MGMT* promoter methylation analysis. All tumour specimens analysed in the present study were obtained by either complete (*n* = 17/35; 49%) or partial (*n* = 18/35; 51%) debulking surgery.

### DNA extraction and bisulfite treatment

To ensure high tumour DNA content, FFPE tissue sections were stained with H&E and histologically examined by an experienced neuropathologist (C. Hartmann). Sections showing a tumour cell content of more than 80% were directly subjected to DNA extraction, while on sections with adjacent non-neoplastic tissue, the tumour portion was microdissected and further processed. Extraction of genomic DNA was performed using the QIAamp DNA Mini Kit (Qiagen, Hilden, Germany) and quantified with a NanoDrop ND-1000 (PeqLab, Erlangen, Germany). Five hundred nanograms of extracted DNA as well as CpGenome Universal Methylated DNA (Chemicon International, Temecula, CA) and CpGenome Universal Unmethylated DNA (Chemicon International) as controls were subjected to bisulfite treatment using the EpiTect Bisulfite Kit (Qiagen). The bisulfite-treated DNA was used for MSP and PSQ, while MS-MLPA was performed with untreated genomic DNA. The efficiency of the bisulfite conversion was checked by analysing the control DNA by pyrosequencing.

### Methylation-specific polymerase chain reaction (MSP)

The two primer sets established by Esteller *et al.* for MSP of *MGMT* [2] were 5′-TTTCGACGTTCGTAGGTTTTCGC-3′ (forward primer) and 5′-GCACTCTTCCGAAAACGAAACG-3′ (reverse primer) for methylated template detection (M primers, product length 81 bp; Figure 1, purple bars) and 5′-TTTGTGTTTTGATGTTTGTAGGTTTTTGT-3′ (forward primer) and 5′-AACTCCACACTCTTCCAAAAACAAAACA-3′ (reverse primer) for unmethylated template detection (U primers, product length 93 bp; Figure 1, mint bars). The PCR was performed in a total volume of 20 µl containing 10 µl HotStarTaq Mix (Qiagen), 1 µl of the respective forward and reverse primer (10 pmol), 6 µl high purity water and 2 µl bisulfite-treated template DNA. The PCR programme was 95°C for 15 min, then 35 cycles of 95°C for 50 s, 59°C for 50 s and 72°C for 50 s, followed by a final step at 72°C for 10 min. PCR reactions with CpGenome Universal Methylated DNA (Chemicon International), with CpGenome Universal Unmethylated DNA Vial A (Chemicon International), and without any DNA (non-template control) were included as controls. PCR products were separated on a 2% agarose gel. For a qualitative assessment, a visible M primer band indicated a positive methylation status, whereas absence of an M primer MSP product was evaluated as a negative methylation status of the respective tumour specimen. For an alternative semi-quantitative approach, images of the agarose gels were analysed with ImageJ software (National Institutes of Health, Bethesda, MD; http://rsb.info.nih.gov/ij). For each specimen, the optical band intensities of the corresponding M primer and U primer MSP products were quantified and corrected against the background of the gel. Tumour specimens with an M/U ratio>1 were assessed as strongly methylated, whereas an M/U ratio between 0 and 1 indicated weak promoter methylation. Tumour specimens with an M/U ratio = 0 (no M primer MSP product) were assessed as unmethylated.

### Pyrosequencing (PSQ)

PSQ was performed on a PSQ 96ID system (Qiagen) with a set of primers provided with the PyroMark MGMT Kit (Qiagen). The primer set covers a region of the *MGMT* promoter located at the start of the first exon (Figure 1, dark blue box), which is adjacent to the region that is covered by the MSP primers. The PCR was performed in a volume of 40 µl containing 20 µl HotStar Taq Mix (Qiagen), 1 µl of each PCR primer (10 pmol), 8 µl high purity water and 10 µl of bisulfite-treated template DNA. The PCR cycling programme for both primer sets was composed of an initial activation step at 95°C for 15 min, followed by 40 cycles of denaturation at 95°C for 30 s, annealing at 53°C for 30 s and elongation at 72°C for 30 s. The programme was finished by a final elongation step at 72°C for 10 min. PCR products were visualized by gel electrophoresis, and 30 µl were subjected to the PSQ sample preparation process. DNA was mixed with streptavidin-coated sepharose beads, followed by strand separation and washing utilising the vacuum prep tool (Qiagen). The single-stranded DNA bound to the sepharose beads was mixed with 40 µl of 0.4 µM sequencing primer solution, heated to 80°C for 60 s and then cooled down to room temperature for annealing. For the sequencing reaction PyroMark Gold reagents were used (Qiagen). The sequencing results were analysed using the PSQ PyroMark software (Qiagen). As controls, CpGenome Universal Methylated DNA (positive methylation control; Chemicon International) and DNA from FFPE non-tumourous brain tissue (negative methylation control) were included in the assay, as well as a reaction without any template DNA (non-template control). Pyrograms of the control DNA were analysed to confirm complete bisulfite conversion. All tumour and control specimens were measured in triplicates.

### Methylation-specific multiplex ligation-dependent probe amplification (MS-MLPA)

Two hundred nanograms of non-bisulfite treated DNA were subjected to the MS-MLPA procedure using the ME011 kit (MRC Holland, Amsterdam, Netherlands). Three GCGC *Hha*l sites within the *MGMT* promoter that are addressed by this kit are depicted in Figure 1 (light blue boxes). The amplification products were separated by capillary electrophoresis on an ABI 3100 Genomic Analyser (Applied Biosystems, Foster City, CA). As reference samples, untreated DNA from FFPE non-tumourous brain tissue was used. The data analysis was performed with SeqPilot 3.3 software (JSI Medical Systems, Kippenheim, Germany).

### Statistical analysis

The prognostic value of *MGMT* methylation data obtained by MSP, PSQ or MS-MLPA was assessed with respect to PFS and OS. PFS was used as the primary read-out as it provided the most valid information on the biological activity of dose-intensified TMZ tested upfront in UKT-05 without being biased by *MGMT*-independent effects of second-line treatments. Survival analysis on an intention-to-treat basis in UKT-05 required initiation of the trial treatment. Survival curve estimation was done using the Kaplan-Meier method [34]. Analysis of the original continuous methylation data was performed using Cox proportional hazards regression models. Missing values were imputed by single imputation applying predictive mean matching using the R package Hmisc, version 3.8–3. Maximally selected log-rank statistics were used to test for cut-points in continuous CpG methylation data obtained by PSQ and MS-MLPA and, if significant, to estimate the corresponding cut-off value [35]. This was done using R package coin, version 1.0–18. Methylation data obtained by PSQ reflect the median of triplicates for each CpG site addressed. *P*<0.01 was considered to indicate statistical significance.

For correlation analysis, partial correlations were computed using the estimation procedure described by Schäfer and Strimmer [36] and implemented in the R package GeneNet, version 1.2.4. To assess the additional prognostic value of methylation measures beyond clinical factors, models with and without MSP, PSQ, or MS-MLPA data were compared by likelihood ratio tests. The model including the major therapy-independent clinical prognostic factors, age, gender, Karnofsky performance status and extent of resection was compared with the models including dichotomised methylation factors obtained by PSQ and MS-MLPA. To assess the predictive accuracy of models including methylation data, the cumulating prediction error curves over 18 months follow-up time (which was near to the median follow-up of 21.7 months) and a time-dependent R^2^-like measure were computed using 10-fold cross-validation taking the estimation of cut-points into account [37]. All statistical analyses were computed using the statistical software environment R, version 2.12.2 [38].
